# Recent Developments in Metabolomics Studies of Endophytic Fungi

**DOI:** 10.3390/jof8010028

**Published:** 2021-12-29

**Authors:** Kashvintha Nagarajan, Baharudin Ibrahim, Abdulkader Ahmad Bawadikji, Jun-Wei Lim, Woei-Yenn Tong, Chean-Ring Leong, Kooi Yeong Khaw, Wen-Nee Tan

**Affiliations:** 1 Chemistry Section, School of Distance Education, Universiti Sains Malaysia, Penang 11800, Malaysia; kashvintha18@gmail.com; 2 School of Pharmaceutical Sciences, Universiti Sains Malaysia, Penang 11800, Malaysia; baharudin.ibrahim@usm.my (B.I.); a.bawadkji@yahoo.com (A.A.B.); 3 Department of Clinical Pharmacy & Pharmacy Practice, Faculty of Pharmacy, Universiti Malaya, Kuala Lumpur 50603, Malaysia; 4 Department of Fundamental and Applied Sciences, HICoE-Centre for Biofuel and Biochemical Research, Institute of Self-Sustainable Building, Universiti Teknologi PETRONAS, Seri Iskandar 32610, Perak Darul Ridzuan, Malaysia; junwei.lim@utp.edu.my; 5 Drug Discovery and Delivery Research Laboratory, Malaysian Institute of Chemical and Bioengineering Technology, Universiti Kuala Lumpur, Alor Gajah, Melaka 78000, Malaysia; wytong@unikl.edu.my (W.-Y.T.); crleong@unikl.edu.my (C.-R.L.); 6 School of Pharmacy, Monash University Malaysia, Jalan Lagoon Selatan, Bandar Sunway 47500, Malaysia; khaw.kooiyeong@monash.edu

**Keywords:** metabolomics, endophytic fungi, bioactive metabolites, nuclear magnetic resonance, liquid chromatography–high-resolution mass spectrometry, extraction, analytical tool, systems biology

## Abstract

Endophytic fungi are microorganisms that colonize living plants’ tissues without causing any harm. They are known as a natural source of bioactive metabolites with diverse pharmacological functions. Many structurally different chemical metabolites were isolated from endophytic fungi. Recently, the increasing trends in human health problems and diseases have escalated the search for bioactive metabolites from endophytic fungi. The conventional bioassay-guided study is known as laborious due to chemical complexity. Thus, metabolomics studies have attracted extensive research interest owing to their potential in dealing with a vast number of metabolites. Metabolomics coupled with advanced analytical tools provides a comprehensive insight into systems biology. Despite its wide scientific attention, endophytic fungi metabolomics are relatively unexploited. This review highlights the recent developments in metabolomics studies of endophytic fungi in obtaining the global metabolites picture.

## 1. Introduction

Endophytic fungi are highly diverse microorganisms that inhabit intracellular and intercellular plant tissues. According to Rashmi et al. (2019), there are approximately one million endophytic fungi [1]. Their wide appearance and distribution in the natural ecosystem indicate the importance of ecological and evolutionary fungi [1,2,3]. Endophytic fungi offer significant advantages to the host plants by producing various metabolites to counter the attack by pathogens, insects, and herbivores. They are a promising group of microorganisms that produce plant-associated bioactive metabolites with diverse chemical entities and structural functions [4,5,6]. Metabolites isolated from endophytic fungi exhibited various pharmacological properties, such as antimicrobial, anticancer, antioxidant, anti-inflammatory, and antidiabetic activities. For instance, Taxol is one of the clinical anticancer drugs produced by endophytic fungi used in the treatment of human cancer diseases [7,8]. Nevertheless, there are challenges in the isolation of bioactive metabolites from endophytic fungi due to the high complexity of the crude extracts. The traditional extraction techniques are laborious and slow. Occasionally, some bioactive metabolites are detected in minor and trace quantities. Thus, metabolomics has emerged as an indispensable approach in the comprehensive analysis of complex crude samples [9,10].

**Figure 1:**
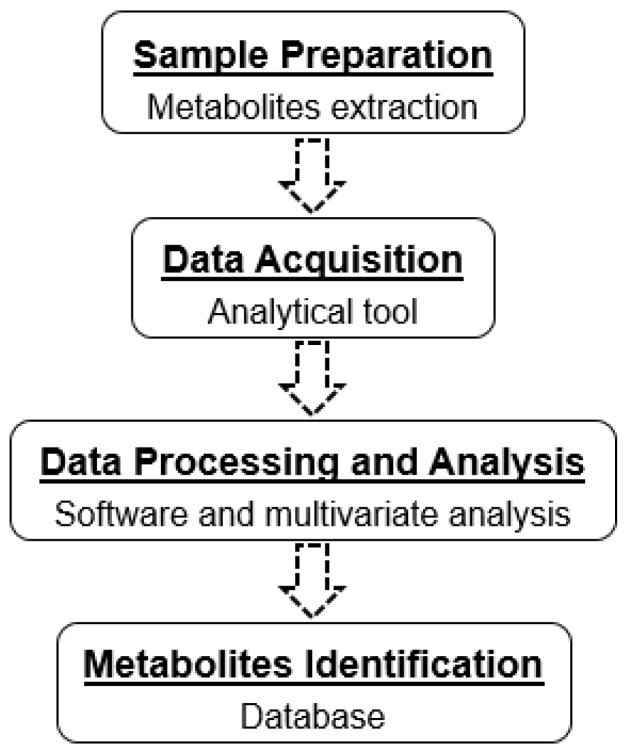
A workflow in metabolomics studies of fungal endophytes.

Metabolomics is an emerging research field that utilizes technological advances in analytical chemistry to measure and compare the metabolites present [11,12]. Nonetheless, the vast chemical diversity of metabolites has posed challenges in data analysis and interpretation. The enormous metabolomics data obtained from analytical tools require multivariate analysis in classifying different sample groups and metabolites distribution when subjected to different experimental parameters. Therefore, advances in analytical tools coupled with multivariate analysis allow the overview of the metabolites presented aided with visual representation. Principal component analysis (PCA), partial least square discriminant analysis (PLS-DA), and orthogonal partial least square discriminant analysis (OPLS-DA) are among the primary analyses used for this function [9,13]. Generally, metabolomics studies start with study design, followed by sample preparation, data acquisition using analytical tools, and then proceed with data processing and analysis. Lastly, metabolites are identified using the established databases (Figure 1) [11,14]. To date, the most commonly used analytical tools in metabolomics studies are mass spectrometry (MS) coupled with liquid chromatography (LC) and nuclear magnetic resonance (NMR). In metabolomics studies, analytical reproducibility is considered one of the most important criteria. Multiple replicates are recommended to account for the effects and/or variations during the data acquisition [12,15].

Endophytic fungi have been regarded as a less exploited niche despite their substantial roles in plants’ protection and drug discovery [16,17]. The present review focused on the recent developments in the metabolomics studies of endophytic fungi isolated from host plants. In the following sections, contemporary metabolites extraction from endophytic fungi is described and discussed. Advanced analytical tools, in particular, liquid chromatography–high-resolution mass spectrometry (LC-HRMS) and NMR are reviewed in relation to metabolomics of endophytic fungi (Table 1). In addition, various experimental parameters, such as fungal culture media, time-based incubation, and analysis, as well as bioactivity of fungal extracts that affect the metabolomics data, are also discussed. Perspectives pertaining to the prospects and challenges of metabolomics studies in endophytic fungi are then concluded.

## 2. Multivariate Analysis

Complications in data analysis in metabolomics studies have posed challenges to researchers. Multivariate analysis plays an important part in mining the key metabolites information from the vast raw dataset [25]. PCA is the most widely used multivariate analysis in metabolomics studies. It is employed to observe the distribution pattern of the sample in a general view along with/without the outliers. Meanwhile, partial least squares (PLS) are used to detect the correlations between variables. The quality and effectiveness of the statistical model are then validated using PLS-DA by obtaining the predictable variables and degree. OPLS-DA is then applied to maximize the variations between different groups in the model. In addition to that, statistical significance using analysis of coefficient is performed to summarize the metabolites information [26,27]. In metabolomics studies, the selection of appropriate multivariate analysis is crucial in achieving the experimental goals. The multivariate analysis provides an essential platform to understand the complex metabolites information in a metabolic system [28].

## 3. Metabolites Extraction

Metabolites extraction from endophytic fungi plays a fundamental role in obtaining a comprehensive metabolites profile [29]. Before metabolites extraction, the isolation of the endophytic fungus from the host takes place. It involves the combination of the sterilized tissue from endophytic fungus with the plant tissue and streaking onto nutrient agar. Occasionally, small sterilized tissues could be plated on nutrient agar [30,31]. Fungal fermentation is followed in either solid-state fermentation (SSF) or submerged fermentation (SMF). Generally, SSF is conducted on a solid substrate. It requires low energy but produces a high concentration of metabolites. SSF has a low water content due to water absorption by the solid substrate. Thus, it enhances the oxygen transfer for the growth of microorganisms. Rice straw, rice hull, and sugarcane bagasse are among the common substrates used in SSF [32,33]. Meanwhile, SMF is a simple fermentation process occurring in excess of water content. Fungi are grown on soluble and/or insoluble substrates, which are submerged in liquid nutrient media. It is widely used due to its better control of fermentation parameters. The pH, temperature, types of culture media, dissolved oxygen, etc., in SMF, could be altered to obtain a wide variety of metabolites [34,35].

Subsequently, metabolites are extracted using appropriate organic solvents such as chloroform, dichloromethane, or ethyl acetate. A suitable and optimized extraction technique is vital to obtain a full profile of metabolites [36]. Liquid–liquid extraction (LLE) and ultrasonic-assisted extraction (UAE) are among the popular techniques used in the preparation of endophytic fungal extract for metabolomics studies. LLE, or known as solvent extraction and partitioning, involves the transferring of metabolites from aqueous to organic solvents by phase separation. This technique separates the metabolites based on solubility in two different immiscible liquids. Separation funnel extraction is the conventional way of performing LLE [37,38,39]. On the contrary, UAE employs high frequency to produce cavitation, providing better penetration between sample and solvent during the extraction process. The ultrasound waves increase the extraction efficiency by disrupting cells for effective mass transfer. UAE is capable of shortening the extraction duration to achieve the ideal extraction efficiency [37,40].

## 4. Advanced Analytical Tools for Metabolomics Studies

Analytical tools that are primarily used in metabolomics studies are LC-HRMS and NMR spectroscopy. They possess their own strengths and limitations in metabolomics studies. It is essential to select the right analytical tool to detect metabolites of interest. The choice of the analytical tool is fundamentally relying on the nature of the metabolomics samples as well as the essence of the study [15,41,42]. Developments and challenges of LC-HRMS and NMR in the metabolomics studies of endophytic fungi are highlighted in the following sections.

### 4.1. LC-HRMS-Based Metabolomics

LC-HRMS is a versatile analytical tool that employs the separation technique of LC with high-resolution mass spectrometry (HRMS) in measuring the mass-to-charge (*m/z*) ratio of metabolites. In order to produce the metabolites’ peak signals for identification, ionization occurs within the metabolomics sample when injected into the instrument. Electrospray ionization (ESI) is among the common ionization methods used in LC-HRMS-based metabolomics. Meanwhile, the high resolution of mass enables the separation or differentiation of metabolites with identical nominal mass but distinct elemental compositions [15,41,43].

Over the past decades, LC-HRMS-based metabolomics studies in endophytic fungi have increased rapidly, accounting for ~80% of the published literature. The rise has indicated the unique strengths of LC-HRMS-based metabolomics. Generally, LC-HRMS is highly sensitive and selective. It can detect samples with concentrations up to nanomolar (nM) with extensive coverage of metabolites. It is a superior tool for targeted and non-targeted metabolomics studies. Another merit in employing LC-HRMS-based metabolomics is the large access to the spectral databases, which is the key to metabolites identification [44,45,46]. For instance, the database MS-Finder has provided more than 290,000 and 35,000 spectral libraries for positive and negative mode MS, respectively [47,48]. METLIN is among the major electronic databases used in LC-HRMS-based metabolomics. It consists of MS/MS data from positive and negative modes collected at three collision energies (10, 20, and 40 V). Comprehensive high-resolution MS/MS spectral data have allowed the researchers to access the spectral resources for metabolomics studies freely. Furthermore, databases such as AntiBase, ChEBI, Dictionary of Natural Products (DNP), Drugbank, FooDB, KNApSaCK, MarineLit, NANPDB, and National Institute of Science and Technology (NIST) have developed rapidly over the past decades [49,50].

**Figure 2:**
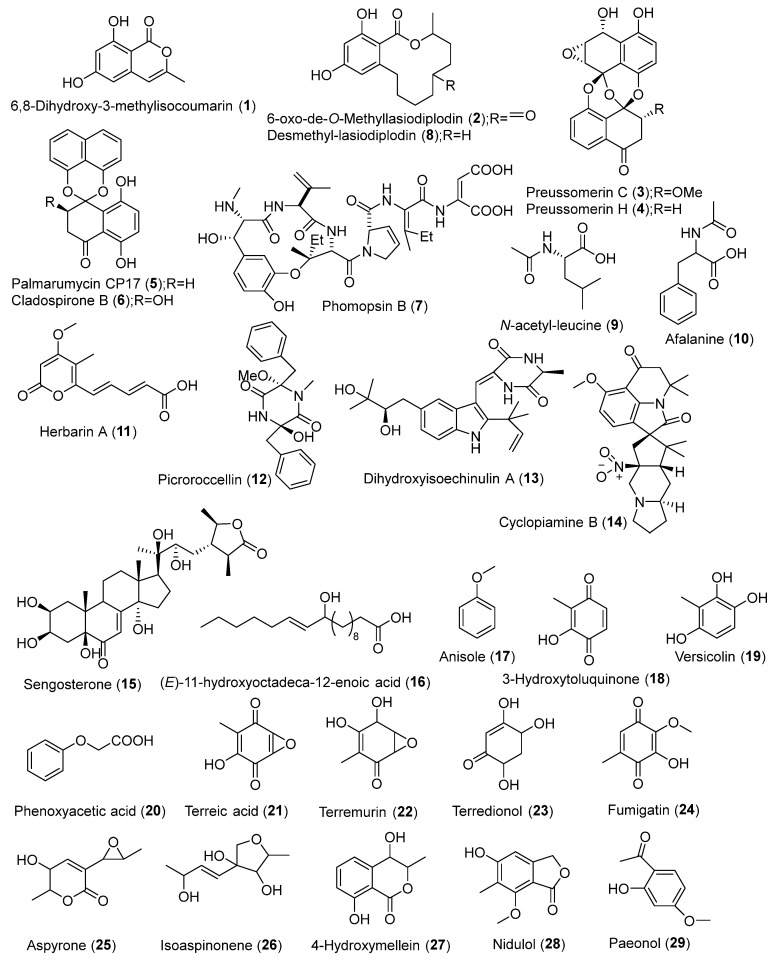

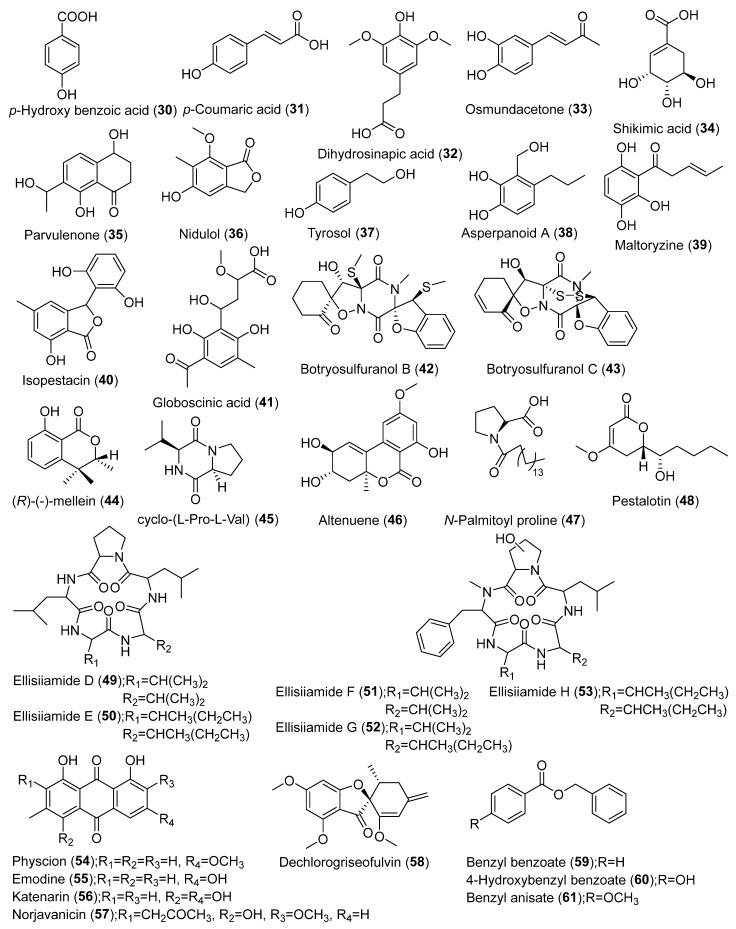
Chemical structures of metabolites (**1**)–(**61**).

Metabolomics studies of endophytic fungi using different culture media and incubation periods are gaining attention in the mining of bioactive metabolites [51,52]. Endophytic fungi *Lasiodiplodia theobromae* grown in liquid and rice media for 7-, 15-, and 30-day incubation were studied in correlation to anti-trypanosomal activity. Based on the results, 7- and 15-day incubation extracts provided a similar chemical profile while a 30-day incubation extract yielded lesser metabolites. However, a bioactivity assay revealed a 30-day rice medium incubated extract exhibited the lowest minimum inhibitory concentration (MIC). Bioactive metabolites 6,8-dihydroxy-3-methylisocoumarin (**1**), 6-oxo-de-*O*-methyllasiodiplodin (**2**), preussomerins-C and H (**3**-**4**), palmarumycin CP17 (**5**), cladospirone B (**6**), phomopsin B (**7**), and desmethyl-lasiodiplodin (**8**) (Figure 2) were identified in the study using OPLS-DA model [18]. Tawfike and co-workers performed metabolomics profiling on *Curvularia* sp. extracts from liquid and rice media on 7-, 15- and 30-day incubation periods in correspondence to cytotoxicity. The findings revealed that 7- and 30-day incubation extracts produced more metabolites than a 15-day incubation extract. A 15-day fungal incubation was regarded as an intermediate phase where metabolites were consumed for survival to counter environmental stress. Different chemical profiles were noticed over the incubation period as different metabolites were synthesized and depleted for different mechanisms. Mass spectral dereplication showed that *N*-acetyl-leucine (**9**), afalanine (**10**), herbarin A (**11**), picroroccellin (**12**), dihydroxyisoechinulin A (**13**), cyclopiamine B (**14**), sengosterone (**15**), and (*E*)-11-hydroxyoctadeca-12-enoic acid (**16**) were detected as unique bioactive metabolites during the growth phase [19]. In a study conducted by Attia et al. (2020), *Aspergillus ochraceus* MSEF6 isolated from *Medicago sativa* was grown in different media, namely, potato dextrose broth (PDB), Sabouraud broth (SAB), malt extract broth (MEB), and rice extract broth (REB) to discover its most potent antimicrobial activity. Fungal extract cultured in PDB was found as the most active with a minimum inhibitory concentration (MIC) of 15–30 mg/mL. Metabolomics-based chemical profiling revealed anisole (**17**), 3-hydroxytoluquinone (**18**), versicolin (**19**), phenoxyacetic acid (**20**), terreic acid (**21**), terremurin (**22**), terredionol (**23**), fumigatin (**24**), aspyrone (**25**), isoaspinonene (**26**), 4-hydroxymellein (**27**), and nidulol (**28**) were present and may contribute to the observed activity [20]. In another study, endophytic fungi *Aspergillus terreus* isolated from soybean was cultured in PDB, acidified potato dextrose broth (MPDB), SAB, MEB, and REB. The obtained ethyl acetate fungal extracts were then subjected to LC-HRMS to produce 2319 and 1230 peaks in positive and negative modes, respectively. Eighteen metabolites were identified after dereplication using the Dictionary of Natural Products database. The metabolites comprised mainly quinones, isocoumarins, and polyketides. Multivariate data analysis reported that MEB, PDB, and MPDB extracts consisted of characteristic chemical fingerprints compared to other cultured media extracts [21]. Endophytic fungi are stimulated by different growth mediums and culture conditions to produce different metabolites. Optimization of culture parameters, co-culture fermentation, as well as the addition of elicitors may be used to increase the production of bioactive metabolites. Metabolomics plays a crucial role in the search for bioactive metabolites from a specific endophyte at specific conditions and parameters [16,53,54,55].

Chemical fingerprints of fungal endophytes may affect by metabolites extraction methods [5,56,57,58]. George et al. (2019) investigated the metabolites produced by *Penicillium setosum* by employing UAE and LLE techniques. Based on the results, endophytic fungal extracts from both extraction techniques gave an almost similar chromatogram when subjected to liquid chromatography–quadrupole time-of-flight mass spectrometry (LC-Q-ToF-MS). Fourteen metabolites were identified from LLE, while eleven metabolites were detected from UAE. They comprised of chemical classes flavonol, flavone, dihydroflavonol, anthraquinone, coumarin, *Penicillium* metabolites, and other organic compounds [22]. The utilization of suitable extraction methods is vital in discriminating the metabolites profiles of endophytic fungi when subjecting to different extraction methods [5,59,60].

Statistical analyses are indispensable in any research study. Multivariate analysis associated with metabolomics serves as a significant approach in dealing with a large number of datasets produced by multiple experiments [25,61,62,63]. Three endophytic fungi (AFL, AFSt, and AFR) isolated from *Artemisia annua* and *Medicago sativa* were studied for their metabolites profiles using LC-HRMS. It revealed the presence of 682 metabolites in the ethyl acetate extracts of AFL, AFSt, and AFR. Phenolic derivatives paeonol (**29**), *p*-hydroxy benzoic acid (**30**), (Figure 2) *p*-coumaric acid (**31**), dihydrosinapic acid (**32**), osmundacetone (**33**), shikimic acid (**34**), parvulenone (**35**), nidulol (**36**), tyrosol (**37**), asperpanoid A (**38**), maltoryzine (**39**), isopestacin (**40**), and globoscinic acid (**41**) were identified as the major metabolites. Additionally, coumarins, alkaloids, and polyketides were detected, among others. Multivariate analysis employing PCA has discriminated the endophytic fungal extracts into three different clusters, indicating their distinctive chemical fingerprints. The PLS-DA-derived heat map displayed the abundance of phenolics in AFL and coumarins in AFSt. Meanwhile, polyketides and alkaloids were predominant in the extract of AFR. The findings are in accordance with the strongest antioxidant activity shown by AFL, followed by AFSt and AFR [1]. Triastuti and colleagues recently worked on a co-culture endophytic fungi *Cophinforma mamane* and *Fusarium solani* in a time-series metabolomics study. By using ultra-high-performance liquid chromatography–high-resolution mass spectrometry (UHPLC-HRMS), 120 and 108 metabolites were identified from positive and negative ionization modes, respectively. Differences in metabolites profile were observed over time (3, 5, and 10 days) in the endophytic fungal extracts of monoculture as well as co-culture. Multivariate analysis using PLS-DA has revealed 25 metabolites that contributed to the group discrimination. Among them, botryosulfuranols B and C (**42**-**43**), cyclosporins A and E, (*R*)-(-)-mellein (**44**), cyclo-(L-Pro-L-Val) (**45**), and cyclo-(L-Leu-L-Leu-D-Leu-L-Leu-L-Val) were identified. In detail, the Venn diagram was used to analyze the metabolites variation in the monoculture and co-culture of fungal extracts. Generally, the number of metabolites increased over time in both monoculture and co-culture of fungal extracts. It is worth noting that co-culture endophytic fungal extract has induced five de novo metabolites, of which three were identified as altenuene (**46**), *N*-palmitoyl proline (**47**), and pestalotin (**48**). Hence, the findings indicated that fungal co-culture in the time-series analysis is worth exploring by researchers in an attempt to discover interesting metabolomes as well as the biosynthetic pathways [23]. Ibrahim and co-workers investigated *Xylaria ellisii*, a new endophytic fungus from *Vaccinium angustifolium*. Fungal filtrates and mycelium from *V. angustifolium* grown in wild and highbush were studied. The ethyl acetate fungal filtrate extract and methanol/acetone (1:1) mycelium extract were profiled using LC-MS-based metabolomics. Nineteen metabolites were identified, consisting mainly of nonribosomal peptides and polyketides. Ellisiiamides D–H (**49**–**53**) were detected as outliers in the extracted filtrates and mycelium employing the OPLS-DA model [13]. In a recent study on endophytic fungi isolated from *Artemisia annua*, antimalarial metabolites were identified using LC-HRMS-based metabolomics. Eleven endophytic fungal isolates from the family of Trichocomaceae, Nectriaceae, and Pleosporaceae were fermented and extracted with ethyl acetate via the UAE technique. Among the 2363 peaks detected in LC-HRMS, eight metabolites were identified. Physcion (**54**), emodine (**55**), katenarin (**56**), norjavanicin (**57**), dechlorogriseofulvin (**58**), benzyl benzoate (**59**), 4-hydroxybenzyl benzoate (**60**), and benzyl anisate (**61**) were found to be positively correlated with the anti-plasmodial activity using multivariate analysis. In further investigation, neural network and deep learning-based software were employed to identify metabolites with the most possible active hits [9]. By applying multivariate analysis in metabolomics, metabolites of interest could be systematically detected and identified from a complex mixture of chemical compounds [64,65,66].

### 4.2. NMR-Based Metabolomics

NMR spectroscopy is a non-destructive, strongly quantitative, and reproducible analytical tool. Owing to its robustness, NMR is automatable, has high throughput, and requires simple sample preparation [67,68,69]. Among the various NMR experiments, one-dimensional proton-NMR (1D ^1^H-NMR) is broadly employed in metabolomics due to its high intense NMR signals and short collection time. The ^1^H-NMR spectrum could be obtained within a minute, and it is well suited for large-scale sample analysis. Furthermore, different nucleic experiments (^13^C, ^15^N, and ^31^P) could be used in NMR to study different chemical classes of metabolites [44,45,70]. Despite its numerous advantages, there are challenges in performing NMR-based metabolomics. NMR is 10–100 times less sensitive compared to MS-based metabolomics. More often, overlapping peaks in ^1^H-NMR spectra have become a great challenge in the characterization of metabolites. Limited NMR databases and software have restricted NMR in metabolomics applications [44,45,71]. Thus, the literature studies employing solely NMR-based metabolomics in endophytic fungi are rather scarce.

Metabolomics investigations using ^1^H-NMR on endophytic fungal isolates (*Colletotrichum* sp., *Diaporthe* sp. and *Periconia* sp.) from *Crescentia alata* Kunth discriminated the classes of compounds present in each extract. The multivariate PLS-DA revealed the strongest anti-inflammatory activity was exhibited by *Colletotrichum* extract, followed by *Diaporthe* and *Periconia* extracts. Chemical shifts δ_H_ 0.76, 1.2, 1.44, 1.72, and 1.76 ppm were noticed in *Colletotrichum* extract, suggesting the presence of terpene-type metabolites. These metabolites were believed to contribute to the observed activity in the *Colletotrichum* extract. Meanwhile, olefinic and aromatic protons were detected in the *Periconia* and *Diaporthe* extracts, respectively. The presence of characteristic NMR signals among the extracts has discriminated the chemical profiles of fungal endophytes isolated from the same host [24]. NMR chemical shift databases could potentially target a list of metabolites hits to the peaks generated from metabolomics experiments. Nonetheless, selecting the right metabolite could be challenging when dealing with metabolites of low concentration. Moreover, the peak assignment becomes complicated when some of the NMR peaks are partly and/or entirely overlapped by highly concentrated metabolites signals [72,73,74,75].

## 5. Conclusions and Future Perspectives

Endophytic fungi are promising producers of biologically interesting metabolites. The bioactive metabolites play a vital role in the treatment and enhancement of human health. Occasionally, potent bioactive metabolites are present at low yields. Thus, metabolites isolation could be challenging at the preliminary stage of works. As such, metabolomics has appeared as a robust approach to measuring the global metabolites from a minimal amount of samples. Innovations in analytical tools have advanced the progress of metabolomics, particularly in endophytic fungi. In-depth research on endophytic fungi by integrating both MS and NMR is warranted in order to perform high-throughput metabolomics studies with better detection and metabolites identification. Continuous metabolomics research on endophytic fungi must remain to ensure contribution to the chemical database of fungal metabolites is increasing for biomarker discovery.

Though metabolomics of endophytic fungi is still in its developing stage, it is highly prospective that the next 10 years will be impactful by integrating both LC-HRMS and NMR in the study design for direct comparison and correlation of the metabolites data. In addition, statistical tools and software, as well as machine learning and neural networks, may significantly aid the data analysis and visual representation of the metabolomics experiments. Studies involving the mechanisms between host plant–endophyte interaction, genetic manipulations of endophytic fungi, and the modified biosynthetic pathways for natural bioactive metabolites from fungal endophytes could be focused on.

## Figures and Tables

**Table 1:** Metabolites identification in endophytic fungi via LC-HRMS- and NMR-based metabolomics.

Endophytic Fungi	Host Plant	Metabolite Extraction	Solvent Used	Analytical Tool	Database	Metabolites	Ref.
*Aspergillus terreus* (AFL, AFSt, AFR)	*Artemisia annua*, *Medicago sativa*	UAE	Ethyl acetate	LC-HRMS	MarinLit, Dictionary of Natural Products	Paeonol, p-hydroxy benzoic acid, p-coumaric acid, dihydrosinapic acid, osmundacetone, shikimic acid, parvulenone, nidulol, tyrosol, asperpanoid A, maltoryzine, isopestacin, globoscinic acid, 5,7-dihydroxy-4-methylcoumarin, β-methylumbelliferone, hymecromone, 7-hydroxycoumarin, scopoletin, citropten, similanpyrone A, flavipin, gliotoxin, isotryptoquivaline, neoxaline, ochratoxin B, indole-3-acetic acid, phenethylamine, gregatin A, aflatoxin B1, aflatoxin B1 exo-8,9-epoxide, penicillic acid, terrein, physcion	[2]
*Aspergillus terreus*, *A. favus, A. oryzae, Penicillium commune, P. chrysogenum, P. chrysogenum, Talaromyces piophilus, T. piophilus, Fusarium oxysporum, F. nematophilum, Pleosporaceae* sp.	*Artemisia annua*	UAE	Ethyl acetate	LC-HRMS	Dictionary of Natural Products	Physcion, emodin, katenarin, norjavanicin, dechlorogriseofulvin, benzyl benzoate, 4-hydroxy benzyl benzoate, benzyl anisate	[9]
*Xylaria ellisii* sp. nov.	*Vaccinium angustifolium*	LLE	Ethyl acetate; Methanol/acetone (1:1)	LC-HRMS	-	Griseofulvin, dechlorogriseofulvin, cytochalasin D, zygosporin E, epoxycytochalasin D, hirsutatin A, piliformic acid, 2,3-dihydro,2,4- dimethylbenzofuran-7-carboxylic acid, cyclic pentapeptide 1 and 2, xylarotide A, ellisiiamides A-H	[13]
*Lasiodiplodia theobromae*	*Vitex pinnata*	LLE	Ethyl acetate	LC-HRMS	AntiBase, Dictionary of Natural Products	6,8-Dihydroxy-3-methylisocoumarin, 6-oxo-de-O-methyllasiodiplodin, preussomerins-C and H, palmarumycin CP17, cladospirone B, phomopsin B, desmethyl-lasiodiplodin	[18]
*Curvularia* sp.	*Terminalia laxiflora*	LLE	Ethyl acetate	LC-HRMS	Natural Product Database	*N*-Acetyl-leucine, afalanine, herbarin A, picroroccellin, dihydroxyisoechinulin A, cyclopiamine B, sengosterone, (E)-11-hydroxyoctadeca-12-enoic acid	[19]
*Aspergillus ochraceus* MSEF6	*Medicago sativa*	LLE	Ethyl acetate	LC-HRMS	Dictionary of Natural Products, METLIN	Anisole, 3-hydroxytoluquinone, versicolin, phenoxyacetic acid, terreic acid, terremurin, terredionol, fumigatin, aspyrone, isoaspinonene, 4-hydroxymellein, nidulol, aspyrone	[20]
*Aspergillus terreus* GMEF1	*Glycine max* L.	UAE	Ethyl acetate	LC-HRMS	Dictionary of Natural Products	Terreic acid, terremutin, (-)-terredionol, terremutin hydrate, 3-methylorsellinic acid, flavipin, astepyrone, reticulol, (3S,6S)-terramide A, emodin, terrelactone A, aspergiketal, 4-hydroxykigelin, 8-hydroxyquadrone, dihydrocitrinone, aspergillide B1, sulochrin, 3α-hydroxy-3,5-dihydromonacolin L	[21]
*Penicillium setosum*	*Withania somnifera*	UAE/LLE	Dichloromethane: ethyl acetate: methanol (3:2:1)/Ethyl acetate	LC-Q-TOF-MS	METLIN	Kaempferol, quercetin, quercetin acetate, luteolin, dihydroqueretin, dihydromyricetin, quinalizarin, isofraxidin, andrastin D, citromycetin, patulin, 6-deoxyerythronolide B, vanillic acid, 2-dehydro-3-deoxy-darabino-heptonate 7-phosphate (DAHP)	[22]
*Coohinforma mamane, Fusarium solani*	*C. mamane* (from *Bixa orellana* L.), *F. solani* (from *Plantago lanceolata*)	UAE	Dichloromethane, methanol and water	UHPLC-HRMS	Dictionary of Natural Products, SciFinder, MS Finder, Natural Product Database, KNApSAcK, Chemical Entities of Biological Interest (ChEBI), STOFF, The Toxin and Toxin Target Database (T3DB), Northern African Natural Products Database (NANPDB), Drugbank, FooDB, PlantCyc	Cyclosporins A and E, botryosulfuranol C and B, cyclo-(L-Pro-L-Val), (R)-(-)-mellein, cyclo-(L-Leu-L-Leu-D-Leu-L-Leu-L-Val)	[23]
*Colletotrichum* sp., *Diaporthe* sp., *Periconia* sp.	*Crescentia alata* Kunth	UAE/LLE	Methanol/Ethyl acetate	^1^H-NMR	-	Terpenes, phenolics, alkaloids, pigments, steroids, polyketides, glycosides	[24]

Note: UAE = Ultrasonic-assisted extraction; LLE = Liquid–liquid extraction.

## Data Availability

Not applicable.

## Abbreviations

ChEBI	Chemical Entities of Biological Interest
ESI	Electrospray ionization
LC	Liquid chromatography
LC-HRMS	Liquid chromatography–high-resolution mass spectrometry
LC-Q-ToF-MS	Liquid chromatography–quadrupole time-of-flight-mass spectrometry
LLE	Liquid–liquid extraction
MEB	Malt extract broth
MPDB	Acidified potato dextrose broth
MS	Mass spectrometry
*m/z*	Mass-to-charge
MIC	Minimum inhibitory concentration
NIST	National Institute of Science and Technology
NANPDB	Northern African Natural Products Database
NMR	Nuclear magnetic resonance
OPLS-DA	Orthogonal partial least square discriminant analysis
PLS-DA	Partial least square discriminant analysis
PCA	Principal component analysis
PDB	Potato dextrose broth
REB	Rice extract broth
SAB	Sabouraud broth
SSF	Solid-state fermentation
SMF	Submerged fermentation
T3DB	The Toxin and Toxin Target Database
UHPLC-HRMS	Ultra high-performance liquid chromatography–high-resolution mass spectrometry
UAE	Ultrasonic-assisted extraction
1D ^1^H-NMR	One-dimensional proton-NMR
